# A Rare Case of Arthroscopic Lateral Segmental Meniscal Allograft Transplantation (SMALT) and Osteochondral Allograft Transplantation (OCA) of the Lateral Femoral Condyle Augmented With Bone Marrow Aspirate Concentrate (BMAC)

**DOI:** 10.7759/cureus.57843

**Published:** 2024-04-08

**Authors:** Sean M Muir, Alyssa McMandon, Siena C Lombardozzi, James D McDermott

**Affiliations:** 1 Medical School, Edward Via College of Osteopathic Medicine, Spartanburg, USA; 2 Surgery, Edward Via College of Osteopathic Medicine, Spartanburg, USA; 3 Biochemistry, Miami University, Oxford, USA; 4 Orthopedic Surgery, Spartanburg Medical Center, Spartanburg, USA; 5 Sports Medicine, Sports Medicine Institute, Medical Group of the Carolinas, Spartanburg, USA

**Keywords:** bone marrow aspirate concentrate, arthroscopy knee, segmental meniscus tear, osteochondral allograft, meniscus transplant

## Abstract

The meniscus of the knee serves as a crucial load-bearing structure, and its damage can significantly impact weight distribution. In addressing focal meniscal defects, segmental meniscal allograft transplantation (SMALT) emerges as an innovative solution. Here, we detail a case involving a young, active female who underwent SMALT augmented with osteochondral allograft transplantation (OCA) and bone marrow aspirate concentration (BMAC). The patient, a 40-year-old former Division I volleyball player, previously underwent arthroscopic procedures and presented with knee pain alongside complex lateral meniscus tear evident in magnetic resonance imaging (MRI) findings. Initial arthroscopy revealed multiple tears, including segmental deficiency at the posterior horn-body junction and a horizontal cleavage tear. Despite failed attempts at repair due to the meniscal gap, a second-stage lateral SMALT was performed, with the allograft soaked in the patient's BMAC, supplemented with OCA to the lateral femoral condyle. Rehabilitation protocols tailored to both SMALT and OCA were implemented. This represents the first documented instance of lateral SMALT, extending the scope of viable solutions for segmental meniscal deficiencies, and marking a significant milestone in orthopedic practice.

## Introduction

The menisci are two cartilaginous structures that play essential roles in knee kinematics and biomechanics. They serve vital functions in load transmission, shock absorption, joint stability, lubrication, and proprioception [1]. The significance of load transmission was initially demonstrated by Fairbank, who showed that the absence of menisci leads to increased osteoarthritis progression, highlighting the meniscus's role as a protective load-bearing structure [1]. While the distribution of load through the menisci may change during flexion and extension, literature reports that approximately 50% of the load in the medial compartment and 70% in the lateral compartment is absorbed by the medial and lateral menisci, respectively [1]. Meniscal damage alters load distribution and disrupts the natural weight-bearing surface.

The role and importance of the meniscus are extensive, and any meniscal injuries can impair knee function, alignment, and stability. Meniscal healing varies due to the dynamic blood flow and watershed areas. The medial and lateral geniculate arteries supply blood to the periphery of the menisci (red-red zone), while the middle third of the meniscus still receives blood, albeit less than the periphery (red-white zone) [1,2]. The avascular zone, the inner third (white-white zone), receives minimal to no blood flow and, therefore, does not heal [1,2]. The current standard of care for symptomatic patients requiring surgery with a tear in the avascular zone of the meniscus is partial meniscectomy. However, tears involving the middle red-white zone and red-red zone should undergo meniscal preservation [2]. Meniscal preservation options include meniscal repair, meniscal allograft transplantation (MAT), meniscal scaffolding transplantation (MST), and segmental meniscal allograft transplantation (SMALT) [3-6]. The choice of meniscal preservation option depends on the patient's age, activity level, the type of meniscal tear, and the surgeon's familiarity with the technique. In this study, we present a surgical technique and a case involving a young, active female who underwent SMALT with osteochondral allograft transplantation (OCA) augmented with bone marrow aspirate concentration (BMAC). We also discuss other novel meniscal preservation techniques, such as MAT and MST.

Surgical criteria**

Medial and Lateral SMALTs are a novel approach to treat focal meniscal defects. The defect may be a result of a prior meniscectomy or an irreparable meniscal tear, resulting in a partial meniscus deficiency with intact posterior and anterior horns. SMALTs should be limited to patients under the age of 50 who present with symptomatic knee pain limiting desired activities and/or classic meniscal mechanical symptoms (popping, clicking, clunking), and have a stable knee exam. Limited evidence of degenerative change, such as an Outerbridge classification of two or less, or an International Cartilage Repair Society classification of three or less, should be noted on magnetic resonance imaging (MRI). If the MRI demonstrates a significant meniscal defect or cartilage degeneration, a SMALT may not be applicable. Other contraindications for SMALTs include significant knee malalignment, ligamentous laxity on examination, and standard surgical contraindications, including, but not limited to, a body mass index (BMI) >35, hyperglycemia >200, or problems with anesthesia. Additional, post-surgical rehabilitation and activity modifications should be discussed with the patient. Any signs of noncompliance with postoperative rehabilitation protocol should be considered as a contraindication.

Preoperative evaluation

Before performing a SMALT a complete medical history and physical examination should be obtained. Patients undergoing a SMALT likely have undergone several previous meniscal surgeries, and therefore, a detailed surgical history is important. Standard knee X-rays (anteroposterior, lateral, and sunrise) are recommended during the initial visit. An MRI is also recommended after a standard physical examination is performed. The evaluation of alignment, meniscal status, chondral lesions, and ligamentous stability should be conducted during the examination and through imaging. Diagnostic arthroscopy may be useful for verifying the grade of chondromalacia and inspecting the cartilaginous surface, as well as the meniscal horns.

## Case presentation

A 40-year-old former Division I volleyball player sought treatment at the sports medicine clinic due to left knee pain that developed four weeks prior. The pain appeared without a specific traumatic incident but was accompanied by significant swelling. She experienced discomfort during everyday activities like walking, crouching, climbing stairs, and getting in and out of bed, which hindered her daily routines and exercise.

Her medical history included left knee pain during her junior year of undergraduate education as a volleyball player. An MRI at that time confirmed a meniscus tear, leading to a surgical intervention that involved a left knee arthroscopy, partial lateral meniscectomy, and chondroplasty, performed by the head team orthopedic surgeon at that institution.

The physical examination revealed mild swelling and a normal range of motion. Ligamentous exams were unremarkable, with no signs of instability. Meniscal exams showed negative findings on the medial side but were positive for lateral joint line tenderness and provocative tests.

An MRI confirmed the integrity of the MCL, ACL, and PCL. Chondral fissuring was observed on the central aspect of the lateral patellar facet articular cartilage. The lateral compartment had a 1.4 x 1.6 cm grade 4 chondral defect on the posterior lateral femoral condyle articular surface. The lateral tibial plateau articular cartilage remained intact with a small focal area of chondromalacia. The lateral meniscus displayed a complex tear, including a radial component on the posterior horn and a horizontal flap tear along the medial meniscus extending into the anterior horn. Other structures, such as the popliteus tendon, fibula collateral ligament, and biceps tendon femoris, remained intact.

Table 1 highlights the indications and contraindications of SMALTS.

**Table 1:** Indications and contraindications for segmental meniscal allograft transplantation (SMALT)

Indications	Contraindications
Segmental defect with intact anterior and posterior horns	Knee malalignment
Active young patients with mechanical or physical symptoms	Ligamentous laxity
Outerbridge < 2 or International Cartilage Repair Society < 3	Noncompliant with rehabilitation expectations

Diagnostic arthroscopy 

After discussion and informed consent, the patient was taken for a diagnostic arthroscopy. Anterolateral and anteromedial portals for standard knee arthroscopy were created using an 11 blade. The arthroscope was inserted in the anterolateral portal into the patellofemoral articulation. The patient demonstrated intact articular cartilage on the undersurface of the patella for the most part with the exception of a small area of grade II chondromalacia measuring 5 x 5 mm. The cartilage within the trochlear groove was perfectly intact. Gentle chondroplasty was undertaken on the undersurface of the patella to remove all loose and unstable cartilage.

The arthroscope was then placed in the suprapatellar pouch, medial and lateral gutters, and multiple intra-articular loose bodies were seen in both the medial and lateral gutters. These were removed with the arthroscopic shaver.

The arthroscope was then placed into the medial compartment where the medial meniscus was probed and was perfectly intact throughout. The medial meniscus roots were probed and intact as well. The medial femoral condyle and medial tibial plateau cartilage were carefully examined and determined to be intact.

Following this, the patient was placed into a "figure of four" position, and the lateral compartment was examined. The articular cartilage of the lateral femoral condyle and lateral tibial plateau was assessed. An evident full-thickness chondral defect was observed on the lateral femoral condyle, measuring about 20 to 25 mm anterior to posterior and 20 mm medial to lateral. The central lateral tibial plateau exhibited grade II chondromalacia, with a localized grade IV chondromalacia area measuring about 5 x 8 mm underneath the segmental deficiency and complex tear of the lateral meniscus.

**Figure 1:**
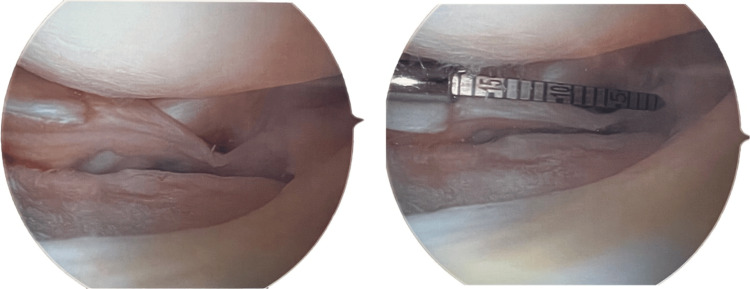
Segmental meniscal deficiency (~15 mm)

Two separate tears were seen in the lateral meniscus, with segmental deficiency at the junction of the posterior horn and body (approximately 10 x 15 mm) due to the patient's prior partial lateral meniscectomy (Figure 1). Additionally, a horizontal cleavage-type tear was observed in the body. Gentle chondroplasty was performed on the lateral tibial plateau and lateral femoral condyle to remove loose and unstable cartilage. The lateral meniscus root and the majority of the posterior horn remained intact, while the body and anterior horn had an exception: the horizontal cleavage tear. This tear was rasped and repaired using an all-inside meniscus repair implant, stabilizing both superior and inferior portions of the tear.

An attempt was made to repair the segmental deficiency with an all-inside meniscus repair implant. However, during the range of motion assessment, redisplacement of the meniscus at the junction of the posterior horn and body revealed the presence of the segmental deficiency (Figure 1). The repair sutures were removed with an arthroscopic biter, and the lateral meniscus body repair remained stable. Chondroplasty was completed, along with the removal of intra-articular loose bodies in the lateral compartment and posterior lateral compartment of the knee.

Meniscal transplantation

The results of the diagnostic arthroscopy were discussed with the patient and the patient consented to a lateral SMALT with OCA of the lateral femoral condyle, supplemented with bone marrow aspirate concentrate (BMAC). When the allograft meniscus and femoral condyle became available, the patient underwent the second stage of the two-stage procedure.

First, 90 cc of bone marrow aspirate was harvested from the patient’s left iliac crest using a Jamshidi needle. The aspirate was processed with the Arthrex® Angel® BMAC system, resulting in 5 ml of bone marrow aspiration concentrate.

The diagnostic arthroscopy then commenced with the creation of an anterolateral portal using an 11 blade. The arthroscope was then inserted and revealed the previous arthroscopic findings: A noticeable meniscal deficiency was observed at the junction of the posterior horn and body, Chondromalacia on the lateral tibial plateau was treated with chondroplasty, primarily consisting of grade 2 chondromalacia, with a small focal area of grade 3/4 measuring 3 x 3 mm, and a substantial osteochondral defect on the lateral femoral condyle, measuring approximately 15 x 25 mm.

The surgeon began the lateral SMALT by measuring the defect in three different locations: the inner third, middle third, and outer third. The defect measured approximately 15 mm on the inner third, 17 mm in the middle third, and 15 mm on the outer third. The defect began about 15 mm from the posterior lateral meniscus root, which was intact, as were most of the lateral meniscus components, including the posterior horn, body, and anterior horn. The segmental deficiency exhibited a somewhat curvilinear pattern. An arthroscopic biter cleaned the meniscal remnant edges, and the lateral meniscus was rasped to promote healing.

Next, with the knee flexed at 90 degrees, a vertical incision of about 4-5 cm was made on the lateral aspect of the knee. The iliotibial band was incised longitudinally, and a popliteal retractor was placed over the lateral capsule.

Inside the knee joint, a long meniscus needle with 2-0 meniscus repair tape was passed into the four quadrants of the peripheral rim of the meniscal deficiency, using zone-specific cannulas. The sutures were managed carefully by the surgical assistant to keep them organized. The arthroscope was switched to the anteromedial portal, and a passport cannula divided into four quadrants retrieved the sutures from each specific quadrant.

**Figure 2:**
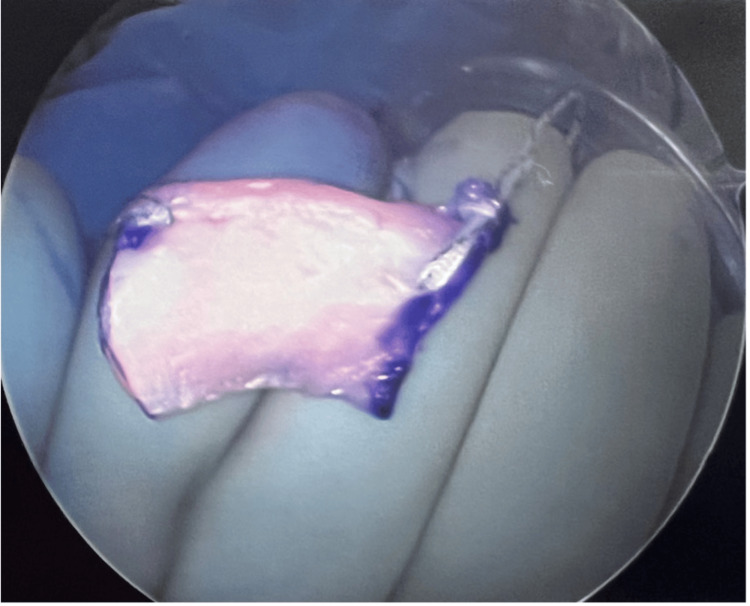
Segmental meniscal allograft transplantation

On the back table, the lateral SMALT was marked and cut to match the patient's deficiency (Figure 2).

**Figure 3:**
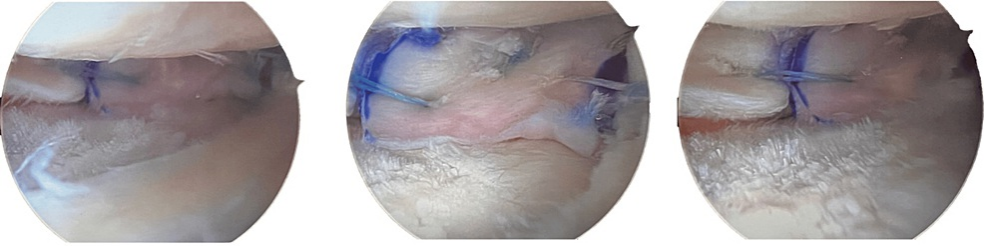
Lateral segmental meniscal allograft transplantation (SMALT)

The allograft was soaked in the patient's BMAC and then injected into the periphery. The allograft was then placed in the knee, and the sutures were used to secure it (Figure 3). All inside meniscus repair implants were used in various locations to stabilize the allograft (Figure 3). The sutures were tied with alternating half-hitch knots. The knee was taken through a range of motion, and the SMALT remained stable (Figure 3).

Osteochondral allograft transplantation

The surgery commenced with a lateral parapatellar arthrotomy, involving a 6-7 cm incision. The defect was exposed, measuring 15 x 27 mm. The size of the defect was properly matched to the appropriate sizing guide.

An osteochondral allograft for the lateral femoral condyle was prepared on the back table and inspected to match the patient's condyle. Marking the graft circumferentially with the appropriately sized guide, followed by scoring and cutting, ensured a good graft fit. The graft was copiously irrigated and soaked in the patient's bone marrow aspiration concentrate.

**Figure 4:**
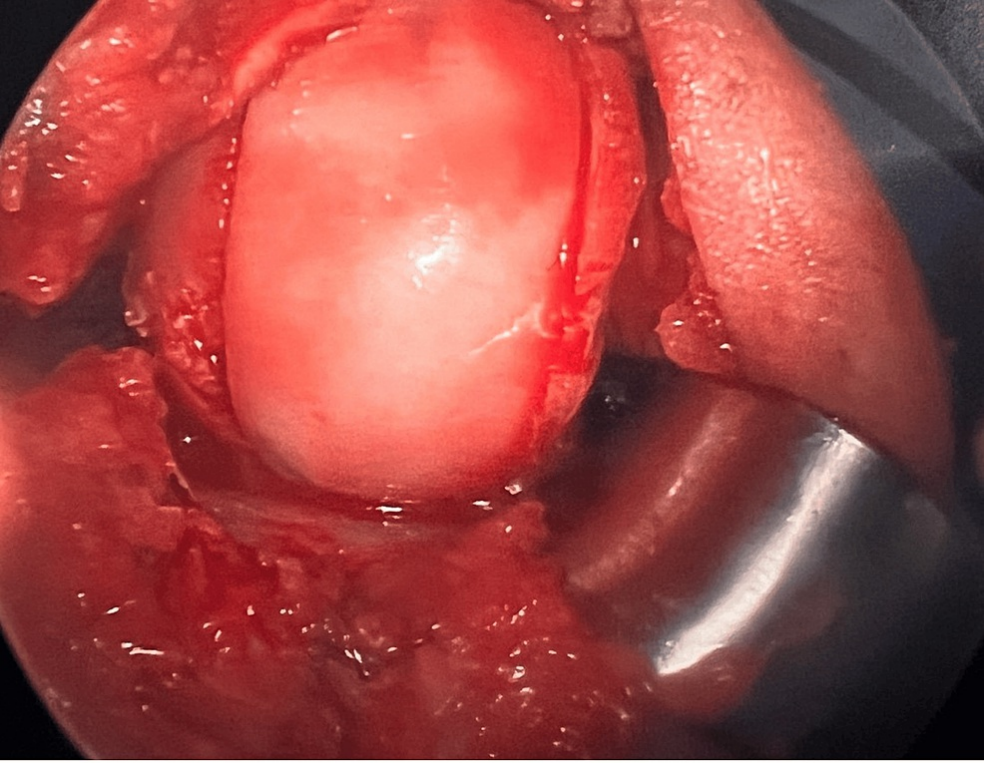
Osteochondral allograft transplantation (OCA)

Returning to the knee, the graft sizing guide was pinned over the defect. The cartilage was scored, and a small inferolateral partial patellectomy was performed for better access. Reaming and cutting followed the placement of the guide. The recipient site was dilated, and the graft was adjusted to match. Microfracture was performed with multiple drill holes placed in the bone at the recipient site. After irrigation and graft placement, stability and range of motion were confirmed (Figure 4). Care was taken to protect the lateral meniscus segmental allograft during hyperflexion.

Closure and postoperative management

The surgical wound was irrigated, and final graft pictures were taken. Closure was performed for the parapatellar arthrotomy, subsequently for subcutaneous tissue, and then for the skin and portals, followed by sterile dressings, ace wrap, TED (thromboembolic deterrent) hose, and a hinged knee brace locked in terminal extension applied. The range of motion was set at 0 to 30 degrees of flexion.

Rehabilitation

The patient followed two protocols: one for SMALT and another for OCA to the lateral femoral condyle. For the first six weeks, the patient had limited weight-bearing to heel touch. During the initial postoperative week, the patient maintained full extension until her first follow-up appointment. Following, the range of motion gradually increased: 0 to 30 degrees from weeks 1-2, 0 to 60 degrees from weeks 2-4, and finally, 0 to 90 degrees from weeks 4-6.

## Discussion

It is well-known that the resection of menisci has detrimental effects on the underlying surface of the knee, increasing articular contact pressure in the tibiofemoral joint and leading to degenerative changes. In 1948, Fairbank was the first to publish on this relationship and the effects meniscectomy can have on the articular surface of the knee [7]. Recent literature has shown that resection of even 20% of meniscal tissue increases contact forces through the articular cartilage by up to 350% [8,9]. Furthermore, the location and volume of meniscal tissue loss have been directly correlated with cartilage degradation [8,9]. Many studies have also reported the importance of menisci for intra-articular neurosensory signaling to the spinal, cerebellar, and central nervous system for conscious perception, which helps balance and stabilize the knee joint [1]. Removing meniscus tissue not only increases contact pressures in the tibiofemoral joint but may also remove mechanoreceptors and other proprioceptive fibers that aid in joint alignment, function, and stability.

Therefore, recent efforts have focused on performing partial meniscectomies as opposed to total meniscectomies; however, long-term outcomes of partial meniscectomies are inferior to meniscal repairs [10]. This shift in focus has led to concentrated efforts on meniscal preservation, including meniscal repairs, meniscal allograft transplantation, meniscal scaffold transplantation, and recently, SMALTs, because of the evidence demonstrating the importance of maintaining meniscal preservation to prevent long-term biomechanical complications [10]. Most algorithms support meniscal repairs as the primary surgical approach; however, the focal meniscal loss may prevent a repair from being possible. As demonstrated in this case, meniscal repair was not possible initially; it was evident that another surgical technique to repair the meniscus was necessary to protect and ensure the longevity of the knee. In this case, due to the shape, size, distribution, and location (not involving the anterior or posterior horn of the lateral meniscus), a SMALT was recommended to the patient. Other options included a MAT or MST.

The patient in this case report represents the ideal candidate for a SMALT. She was a former Division I athlete who sustained a meniscal injury during her junior year in college. After many years following a partial meniscectomy, she started experiencing knee pain, and upon further investigation, was found to have a meniscal tear. She was still under 50 years of age, maintained an active lifestyle, and regularly exercised. She was adamant about improving her condition and was dedicated to the postoperative rehabilitation plan. Meniscal repair was attempted and failed. The anterior and posterior horns of the meniscus were intact, and a large deficiency was noted in the posterior 1/3 of the meniscus. A total MAT was not necessary, and MSTs have shown mixed long-term survivability. Therefore, the patient agreed that a SMALT was the best option.

To the authors' knowledge, this is the first documented lateral SMALT case presentation [11]. Nyland et al. and Haber et al. both investigated the medial mean contact pressures post-segmental medial SMALT in a bovine model and in humans, respectively and concluded that medial SMALT restored the medial compartment mean contact pressure and mean contact area to volume to that of an intact medial meniscus [6,12]. To date, no long-term studies have investigated medial or lateral SMALTs and future studies should focus on long-term viability and outcomes.

Long-term survival analysis on MATs has demonstrated decent results. A review by Novaretti et al. analyzed eleven papers and 688 MATs [13]. The mean survivorship at 10 years was 73.5%, and 60.3% at 15 years, with two studies reporting 19- and 24-year survivorship of 50% and 15.1%, respectively [13]. Postoperative knee outcomes and osteoarthritis scores improved in pain, symptoms, functional daily living, sports and recreation, and quality of life [13]. Surgical technique and concomitant injuries addressed during surgery were also investigated.

Another option for preserving meniscal tissue is the use of Meniscal scaffolds [4,14,15]. Advancements in biologic scaffolding and bioengineering in the twenty-first century have made meniscal scaffolds a viable solution for meniscal deficiencies that cannot be repaired [4,5]. Currently, two primary types of scaffolds are in use: a collagen-based implant, also known as CMI (CMI, Ivy Sports Medicine, Gräfelfing, Germany), and the Actifit polyurethane scaffold (Actifit, Orteq Ltd, London, UK) [5,15].

The long-term results of MSTs have shown variability, but recent findings have been encouraging [15]. Some CMI studies have reported a 10-year survival rate of 85% and even as high as 87.8%, with a 14-year survival rate of 64% [14,15]. The Actifit scaffold has demonstrated a 10-year survival rate of around 80%, with no significant differences in pain, function, or activity ability when compared to CMI [14].

However, it's important to note that while recent studies have shown long-term survivability, there are concerns regarding meniscal scaffold issues such as fragmentation, shrinkage, and extrusion persisting, which may lead to an increased risk of failure [5].

This case report has limitations, including a lack of long-term follow-up data. It may also not be suitable for patients with substantial meniscal deficiencies, who could benefit more from traditional transplants. Specific risks include potential allograft integration issues and theoretical concerns about over-constraining the meniscus.

## Conclusions

Many medial segmental meniscal allograft transplantations (SMALTs) have been documented; however, this is the first documented case of a lateral SMALT. Biomechanical studies have shown that SMALTs can restore articular surface contact pressures similar to a native knee, making them a preferred solution for focal segmental meniscal deficiencies. Other options include meniscal allograft transplantation and meniscal scaffold transplantation.
